# Effectiveness of nurse case management compared with usual care in cancer patients at a single medical center in Taiwan: a quasi-experimental study

**DOI:** 10.1186/1472-6963-13-202

**Published:** 2013-05-31

**Authors:** Yu-Chih Chen, Yu-Jen Chang, Yi-Chen Tsou, Mei-Chuan Chen, Yu-Chu Pai

**Affiliations:** 1 Taipei Veterans General Hospital, Taipei, Taiwan; 2 Department of Health Administration, School of Public Health, University of South Carolina, Taipei, Taiwan; 3 Institute of Community Health Nursing, National Yang-Ming University, Taipei, Taiwan; 4 Graduate Institute of Long-Term Care, National Taipei University of Nursing and Health Sciences, Taipei, Taiwan; 5 Department of Nursing, Fu Jen Catholic University, Taipei, Taiwan; 6 School of Nursing, National Defence Medical Center, Taipei, Taiwan

## Abstract

**Background:**

In order to improve treatment and care quality for cancer patients, nurse case management model has applied generally in the clinical practice. However there were only few evidence-based studies on the relative benefits in Taiwan. Further analysis and feedback application are needed. The aim of this study is to evaluate the effectiveness of care quality in cancer patients with nurse case management.

**Methods:**

This study was conducted with a quasi-experimental design in a national medical center in Northern Taiwan. Patients diagnosed as lung, liver, breast, colon, buccal or cervical cancers were eligible for inclusion. A total number of 600 subjects randomly selected from the cancer case management system enrolled in the case managed group, and 600 patients who received usual care were randomly selected from cancer registry and enrolled in the control group. The study instrument was developed to measure care effectiveness, including the rates of patient continuing treatment, non-adherence to treatment, prolonged hospitalization, unplanned readmission, and planned admission for active treatment. The content validity of expert was assessed as 0.9.

**Results:**

The nurse case management significantly decreased the unplanned readmission rate caused by infection (1.5% vs. 4.7% in the control group, p = 0.002). The rate of patient continuing treatment in the institution significantly increased in the case managed group (93.8% vs. 84.8% in the control group, p < 0.001). The planned admission rates in 14 days and in 15–30 days for active treatment also significantly increased in the case managed group (18.4.% vs. 3.9% in the control group and 34.5% vs. 10.4% in the control group, respectively, p < 0.001). The results indicated that nurse case management provided better control in timeliness and continuity of patient treatment.

**Conclusions:**

This study demonstrated that cancer case management could improve the effectiveness of cancer care services and concretely illustrated a comprehensive model for oncology patients in Taiwan. In addition, the model could be optimized for further application and improvement of cancer care. Future investigations are needed to develop precise and rigorous evaluation to optimize the utilization of cancer case management.

## Background

Cancer has become the leading cause of death in Taiwan since 1982 [1]. The incidence rate of cancer increased dramatically from 111 to 276 per 100,000 people between 1982 and 2008 [2]. The soaring prevalence and incidence urgently call for better management of cancer care.

Advancement in cancer treatments has prolonged the survival time of cancer patients [3]. In Taiwan, the national health insurance system (NHIS) exempts oncology patients from the medical co-payment of cancer care. This results in a heavy burden of medical expenditure and a serious financial deficit to NHIS [4]. Previous studies have demonstrated the benefits of cancer case management on treatment continuity and care quality through the challenging trajectory [5-7]. Nevertheless, the empiric reviews of the case management programs showed heterogeneity in study designs and outcome measures. The effectiveness of nurse case management is thus inconclusive [8-10]. Further studies with clear definitions on the role of management are required to repeat the examinations on clinical efficacy [9].

As nurse case management is a relatively new concept in Taiwan, the application in cancer cases remains to be developed. The aim of this study, therefore, was to evaluate the effectiveness of nurse case management in cancer patients, and to illustrate a concrete model for future implementation in Taiwan.

### The concept of nurse case management

Nurse case management has been widely used to provide high quality care with efficient utilization of medical resources. The system combines essences of patient-centered, multidisciplinary, and organizational care. It integrates health care services, improves completeness of treatment, coordinates resource referral, and is also cost-effective [11].

To perform a holistic care, nurse case managers place great importance on individualized care. They develop partnership and trust with patients, facilitate communication between patients and care providers, and empower patients with knowledge of disease care. They also take responsibility for the supervision, feedback, and evaluation of program implementation and offer valuable insights for referring further application [12,13].

In recent years, cancer patient navigation has been initiated to perform integrated care and eliminate disparities in medical resources among patients of different socioeconomic status and races [13]. Some programs emphasize on overcoming barriers to cancer care, dealing with financial burden, and facilitating health care equity. However, other programs intend to improve timely access of care, patient empowerment, and resource utilization [11]. In spite of the different emphases, these programs aim to improve cancer care based on the concept of case management.

### Effectiveness of nurse case management

The assistance of nurse case management in information gathering, communication, and decision-making indeed improves treatment delivery. Intervention effectiveness was reported in time to diagnosis, time to initiate cancer treatment, and the total numbers of patients treated [7,8,14,15]. It was also reported that patient adherence to cancer care enhanced as well [15]. The improvement in hospital waiting time, doctor availability, resource support, and financial assistance was also noted [5,6].

It has been demonstrated that case management benefits patient empowerment and promotes adjustment to cancer [6]. The needs of physical and psychosocial assessment, adaptation to home environment, and education of illness management account for major proportion of nursing consultation [16]. Lower number of hospitalization, fewer cancer-related problems, better recovery and quality of life support the effectiveness of nurse case management on cancer care [6,17].

Both oncology patients and healthcare providers support the application of navigation services. The overall satisfaction on navigation program was reported to be high [5,6,8]. The results also demonstrated that the navigators facilitate the care delivery [5]. The personality and warmth of navigators play an important role in the success of programs that deliver patient-directed services [15]. Provision of information and emotion support has positive effects on reducing emotional distress and anxiety, in addition to improving perception of body image of the cancer patients [18].

Previous studies revealed that patients with poor social support received greater benefit from nurse case management [16,17]. However, the positive outcomes urged that future interventions be generally applied to all cancer patients rather than limited to those of specific socioeconomic or racial minority [5].

The literature reviewing nurse case management programs has demonstrated positive outcomes in various aspects. However, heterogeneity in the studies, including the diversity in target groups, intervention settings, outcome measures, and methodologies has made it hard to determine the overall effectiveness of case management. Rigorous methods and unequivocal outcome measures are thus required to validate the effects of nurse case management [8-10].

### Nurse case management in Taiwan

The majority of Taiwanese are predominately Han Chinese who speak mandarin and share similar values and cultures. Language barriers, cultural differences, and issues of ethnic minority are less emphasized in the healthcare system. In Taiwan, over 95% people enroll in the national health insurance to receive benefits from health care coverage for general treatments against cancer. Under the sociocultural background, nurse case management in Taiwan focuses on eliminating difficulties in illness trajectory and facilitating treatment completeness, rather than reducing barriers arose from racial and cultural disparities.

Only few studies reported about the effectiveness of nurse case management in Taiwan. For example, a four-year study on the nurse case management in a medical center reported a high satisfaction of cancer patients and medical staffs. The rate of patients lost to follow-up decreased from 13.5% to 1.5%. Patients treated in the institute increased from 95% to 98.1%. The completion rate of treatment plan, frequency of case discussion, treatment adherence, and documentation of side effects were also improved [19].

## Methods

### Study design

This study was conducted with a quasi-experimental 2-group design. The investigation was carried out with cancer patients with nurse case management in 2008. Patients newly diagnosed with lung, liver, breast, colon, buccal or cervical cancers were included in the care program. After one year in the program, the patients receiving case management services were randomly selected as the case managed group. Patients newly diagnosed with cancer in 2007 and receiving usual care for one year were randomly selected from the cancer registry database as the control group. Two groups of 600 patients were compared with each other to evaluate the effectiveness of nurse case management.

### Procedures

This study was conducted in a 3000-bed medical center in northern Taiwan, with 5000~6000 newly-diagnosed cancer cases annually, and the study was performed after the approval of the ethics committee of the institution (Institutional Review Board, Taipei Veterans General Hospital, IRB number: 98-05-12A). Eligible participants were invited by case managers through face-to-face interview. Six hundred randomly selected participants were enrolled and all of them signed the informed consent with a return rate of 100%. Informed consent of the control group was not acquired due to the chart review measure. Every enrolled participant was followed for the study and data were collected from medical records.

### Description of the intervention

Many studies supported oncology nurses to take charge of case management [6,7,11]. Nurse case managers of this program were implemented full time by senior nurses with bachelor degrees and oncology experience for over ten years. They completed an 80-hour training course as a prerequisite, and each case manager was in charge of patients with particular cancer diagnosis according to her own specialty.

The case management services started with the confirmation of cancer diagnosis. Physicians informed the case manager of newly-diagnosed cancer patients. The case managers then contacted the patient face-to-face within 24 hours, introduced their role, and offered consultation by phone open for 24 hours a day should there be any concerns. After the first contact, the case managers carried out in-depth data collection with a cancer specific record chart to assess individual disease information and to understand patients ability of self-care as well as capability of caregivers and support system. They are also responsible to recommend appropriate medical resources following identification of individual needs and barriers.

A multidisciplinary team for nurse case management, integrating expertise of physicians, radiologists, case managers, nurses, nutritionists, physical therapists, pharmacists, respiratory therapists, social workers, home health care nurses, and hospice nursing specialists, met periodically to discuss treatment direction, to evaluate therapeutic effects, and to provide further recommendations. Before a treatment plan was made, the case manager assisted patients and their families to acquire basic knowledge about the disease and treatment, facilitated communication with the multidisciplinary team, and helped the patients to prepare themselves for the potential upcoming difficulties. During the treatment phase, the case manager coordinated the cancer care system, arranged appointments between disciplines, monitored treatment effects and possible complications, provided education and training about disease care and management of side effects, offered emotional support, and improved the adherence for treatment completeness. The case managers visited the patients during every hospitalization to understand their conditions and concerns, and to assess further needs, referrals or resources that might be useful. Before the patients were discharged from the hospital, the case managers discussed further treatment and follow-up plans with the patients and their families, evaluated the patients’ abilities of self-care, assessed household environment and provided telephone counseling open 24 hours a day for possible difficulties.

### Measures

Integrated with the study purpose, relevant literature search and clinical practices [7,19], the survey questionnaire was developed for data collection. It was reviewed by five experienced experts with an overall content validity index of 0.9. The questionnaire consisted of socio-demographic characteristics and information of cancer diagnosis and treatment. We chose the latest hospitalization data (one year after diagnosis) to make comparison for effectiveness evaluation. The selected indicators were: 1. the rate of prolonged (over 30 days by definition of the institution) hospitalization; 2. the rate of non-adherence to treatment, that is, patients refused the recommended therapy or discontinued treatment; 3. the rate of patients to continue treatment in this institute; 4. the rate of unplanned hospital readmission and cause analysis; 5. the rate of planned readmission for active treatment.

### Data analysis

Recorded data were analyzed with SPSS package version 18. The demographic characteristics were presented with percentages, means, and standard deviation. Group equivalence was tested with two-tailed t tests and ANOVA. Proportion comparison of the variables to evaluate effectiveness were examined by chi-square. The statistical significance was defined when *p* value was less than 0.05.

G-power version 3.1 was applied for sample size calculation with a power of 0.80 at an alpha of 0.05. Referring to the literature of nurse case management data in another medical center in Taiwan [19], it was determined that a sample size of 576 cases in each group was necessary. A total of 1200 participants were proposed for potential attrition, with 100 participants in each of 6 different types of cancers diagnosed for the case managed or control group.

## Results

The mean ages of the patients in the case managed and control groups were 60.6±14.0 years and 62.8±13.3 years, respectively. No significant difference in age was found between the two groups, but the case managed group was slightly younger. Some subjects died during the study, including 1.3% patients in the case managed group and 2.8% patients in the control group, but there was no significant difference in the mortality rate between the two groups. Other demographic characteristics of the two groups were also equivalent statistically (Table 1).

The comparison of outcome indicator was illustrated in Table 2. A higher rate of prolonged hospitalization was observed in the case managed group (6.7%) compared with the control group (5.0%). But no significant difference was found.

The rate of non-adherence to treatment was significantly higher in the case managed group

**Table 1:** Comparisons of patient demographics

**Characteristic**	**Experimental group**		**Control group**		***X***^***2***^	***p***
	**(N=600) n %**		**(N=600) n %**			
**Gender**					0.03	.862
Male	286	47.7	289	48.2		
Female	314	52.3	311	51.8		
**Mean age** (years)	60.6 ± 13.96		62.8 ± 13.31			
**Age** (years)					10.56	.061
≤ 45	86	14.3	55	9.1		
46-55	144	24.0	145	24.2		
56-65	151	25.2	160	26.7		
66-75	110	18.3	105	17.5		
76+	109	18.2	135	22.5		
**Marital status**					2.47	.116
Single	30	5.0	43	7.2		
Married	570	95.0	557	92.8		
**Education**					1.28	.258
< High school	313	52.2	332	55.4		
High school	287	47.8	267	44.6		

 (6.5% vs. 1.5% in the control group). Fear for the side effects of the treatment was one important reason for the patients to refuse recommended therapy. Aged patients tended to refuse active treatment such as surgery and chemotherapy, and chose alternative palliative treatments. The reasons for discontinuing treatment included severe complications or side effects of the treatment, recurrent tumor, and occurrence of comorbidity, such as stroke.

The rate of patients continuing treatment in this institute was significantly higher in the case managed group (93.8%) than the control group (84.8%). The reasons for not continuing treatment in this institute included transferring to other hospitals, moving abroad, and lost to follow-up.

The rate of unplanned hospital readmission was lower in the case managed group (5.7%) than in the control group (7.7%) although there was no statistically significant difference between groups was found. As the two most common complications of cancer patients, infection and bleeding were further examined as the causes of unplanned hospital readmission. The rate of unplanned readmission caused by infection was significantly lower in the case managed group (1.5% vs. 4.7% in the control group). The rate of unplanned readmission caused by bleeding was lower in the case managed group (1.2% vs. 1.7% in the control group) but there was no statistical significance.

The rate of planned readmission in 14 days for active treatment was significantly higher in the case managed group (18.4%) than the control group (3.9%). The rate of planned readmission in 15-30 days was also higher significantly in the case managed group (34.5% vs. 10.4% in the control group).

## Discussion

The results of this study supported positive effects of nurse case management in timeliness and frequency of treatment regimen. It reduced unplanned readmission due to complications, improved patients’ reliance on the hospital, and further enhanced treatment continuity of cancer patients.

**Table 2:** Comparisons of outcome indicators

	**Experimental group**		**Control group**		***X***^***2***^	***p***
	**(N=600)**		**(N=600)**			
Indicator	n	%	n	%		
Prolonged hospitalization	40	6.7	30	5.0	1.52	.268
Non-adherence to treatment	39	6.5	9	1.5	19.53	.000***
Continuing treatment in this institute	555	93.8	492	84.8	24.48	.000***
Unplanned hospital readmission	33	5.7	41	7.7	1.92	.165
Cause of unplanned hospital readmission						
Infection	9	1.5	25	4.7	9.40	.002**
Bleeding	7	1.2	9	1.7	0.48	.489
Other	17	2.9	7	1.3	3.35	.067
Planned readmission for active treatment						
In 14 days	107	18.4	21	3.9	56.97	.000***
In 15–30 days	200	34.5	55	10.4	90.64	.000***

*p<.05, **p<.01, ***p<.001.

The rate of patients continuing treatment in the institute and readmission for active treatment was significantly higher in the case managed group. The reasons for readmission were mainly for post-diagnosis surgery and scheduled chemotherapy regimen. These findings were consistent with earlier studies [5,7,14,19]. Nurse case managers promoted patients’ reliance on the medical institute, offered assistance in treatment process, and further improved timely treatment, completion of cancer treatment regimen, and follow-up.

Although cancer progression and complications made patients vulnerable to infection, the rate of unplanned readmission due to infection was significantly lower in the case managed group. It demonstrated the important role of nurse case managers on providing knowledge of disease care. Similar findings were presented in other studies [8,16]. In the treatment period, the case management intervention often focuses on the education of disease related care. In the initial stage of returning home after hospitalization, nurse case managers may help the cancer patients on home care and adaptation.

A higher rate of prolonged hospitalization was observed in the case managed group. Although statistical significance was not found, this result differed from an earlier study which reported a shorter duration of hospitalization for the patients under nurse case management [6]. One possible reason was that the case managed group, with younger patients, might take more invasive treatments which lengthen the hospital stay. Another explanation might be the increasing trend of patient-centered care. With the advancement of treatment, more people received outpatient therapy, and reduced unnecessary hospitalization. However, patients with inconvenient transportation might extend hospitalization to receive treatment regimen. Additionally, the improvement of oncology medicine also prolongs the length of hospitalization for terminal care.

With better understanding of patients’ needs, nurse case managers might facilitate extension of hospitalization to ensure treatment integrity and provide holistic care. Is it in fact that case managers keep patients in the hospital for longer? Although our current data were insufficient to support this viewpoint, it is worthy of future investigation.

Systematic reviews showed that navigation programs improved adherence to cancer care [15]. Unfortunately, we failed to present potential benefits to treatment adherence. In our study, aged patients tended to refuse recommended therapy because of fear of side effects, and chose palliative treatment instead. However, their compliances of palliative regimen were good. Most patients who discontinued treatment were due to involuntary reasons, such as severe complications, recurrent tumor and comorbidity. Some of the patients’ condition became worse during the treatment period, and the nurse case managers referred them to hospice shared care team who provided services that best suited the patients’ conditions [20,21].

The reasons for non-adherence were complicated and deeply influenced by patients’ age, family, and background. The results reported in this study might not be rich enough to present the whole picture. Nurse case managers could recognize changes in patient’s treatment plans and found out the possible underlying reasons for the patients in the case managed group. On the other hand, information of the patients in the control group was acquired by chart review only. It was difficult to identify whether patients followed recommended therapy and the major reasons of non-adherence. This limitation might cause loss of cases and result in a higher rate of non-adherence in the case managed group.

To facilitate the integration of multidisciplinary team and improve the quality of care, the National Health Board of Taiwan offers additional budget for planning and consultation of cancer patient treatment. The study institute did not apply for the grant until this program initiated. At the end of this study, 76.0% of the patients in the case managed group, who had comprehensive treatment protocol and completed the entire treatment, were eligible for the grant. The institute gained extra 32000 US dollars through this program to ease the financial burden and increase resource utilization of cancer care.

### Limitations

This study applied objective indicators from medical records to explore the effectiveness of nurse case management. The data accuracy was not affected by the blindness of study design. The indicators used in this study were based on previous studies [7,19]. Some of the indicators were indirectly measured and were easily influenced by complex factors. Future investigations using different evaluation models are required to identify new determinants that best demonstrate the effectiveness of case management [9].

Because of the research ethics and limitation of clinical practice, this program could not conduct a randomized control study. Although random selection from subject pools was applied to reduce individual differences within the two groups, the lack of control over possible confounders between both groups might diminish the strength of results. Furthermore, the cohort effect resulting from concurrent improvement in oncology medicine, treatment regimen and nursing care practice might influence the effectiveness of the intervention.

To probe general effectiveness of nurse case management, the data of patients with different types of cancer were summed up for analysis. The impact on each particular cancer diagnoses might be thus ignored. Moreover, patients diagnosed with a specific type of cancer were cared by one exclusive cancer case manager. The possible influence derived from individual cancer case managers was difficult to eliminate. However, patients across different kinds of cancer diagnosis held overall positive attitudes toward nurse case management.

### Implications

This study established a model of nurse case management for standard care in cancer patients in Taiwan and contributed to additional financial support from the national health care system. The outcome evaluation and opinions offered by patients during the process served as the valuable references for further implementation. The administration increased the number of cancer case manager from 6 to 14 and included all cancer types in the program. The results could also provide useful information for managing other disease and promote the quality of clinical care.

## Conclusions

Nurse case management in this study demonstrated positive outcome on planning timeliness and completion of treatment regimen. It facilitated better control of the patients’ conditions, improved their reliance on the hospital, and supported them in the process to fight cancer. Further studies are needed to investigate the effects of case management on different cancer types and find out specific needs in particular cancer diagnosis. The fast advancement of clinical medicine increases the diversity of treatment and care programs. Outcome evaluation of nurse case management is recommended to integrate with qualitative approaches to uncover the entire experiences of cancer patients.

## Competing interests

The authors declare no competing interests.

## Authors’ contributions

YCC and YCP supervised and contributed to the study design, coordination, implementation and data interpretation. YJC participated in data collection, analysis and drafted the manuscript. YCT and MCC were responsible for carrying out the study, data collection and analysis. All authors read and approved the final manuscript.

## Pre-publication history

The pre-publication history for this paper can be accessed here:

http://www.biomedcentral.com/1472-6963/13/202/prepub
